# Loading of the Spine in Low Back Pain Patients Does Not Induce MRI Changes in Modic Lesions: A Prospective Clinical Study

**DOI:** 10.3390/diagnostics12081815

**Published:** 2022-07-28

**Authors:** Hanna Hebelka, Helena Brisby, Alfred Erkmar, Kerstin Lagerstrand

**Affiliations:** 1 Department of Radiology, Sahlgrenska University Hospital, 413 45 Gothenburg, Sweden; 2 Institute of Clinical Sciences, Sahlgrenska Academy, University of Gothenburg, 405 30 Gothenburg, Sweden; helena.brisby@vgregion.se (H.B.); guserkal@student.gu.se (A.E.); kerstin.lagerstrand@vgregion.se (K.L.); 3 Department of Orthopaedics, Sahlgrenska University Hospital, 413 45 Gothenburg, Sweden; 4 Department of Medical Physics and Biomedicine, Sahlgrenska University Hospital, 413 45 Gothenburg, Sweden

**Keywords:** Modic change, low back pain, MRI, spinal loading, bone marrow lesion

## Abstract

Modic changes (MCs) are gaining increased interest as potential generators of low back pain (LBP). The current aim was to investigate possible spinal loading effects on the MRI signal in MCs in patients with LBP. Supine lumbar MRIs were performed and immediately repeated with axial loading in 100 LBP patients. A total of 43 patients (23 male, mean age 45.7 years) had MCs. Each Modic was outlined on all sagittal T2-weighted images (>25% affected vertebrae). For reference, regions of interest were placed in both vertebrae without Modic and in Modic-free tissue in vertebrae with Modic. The Modic signal intensity, normalized to cerebrospinal fluid, and Modic volume were compared between MRIs with and without spinal loading. Of the 94 MCs, 36.2% (n = 34) were type I, 58.5% (n = 55) were type II, and 5.3% (n = 5) were type III. No differences in Modic volume (mean 0.046 cm^3^; *p* = 0.25) between the MRIs with and without spinal loading were found. In addition, no significant changes in Modic signal were induced by loading (mean 1.5% difference; *p* = 0.308). Loading increased the signal in the reference regions of interest in vertebrae both with Modic (mean 5.5%; *p* = 0.002) and without (mean 3.5%; SD 0.09; *p* = 0.02). To conclude, MRIs performed with and without spinal loading showed no change in either volume or signal of MCs, suggesting that most MCs are not instantaneously influenced by biomechanical load.

## 1. Introduction

Modic changes (MCs) are a radiological classification of vertebral bone marrow lesions displayed as changes in MRI signal adjacent to the endplates [1,2,3]. In the last two decades, MCs have gained increased interest as potential pain generators in patients with low back pain (LBP) [1,4,5,6]. Although the prevalence of MCs is higher in patients with LBP, these changes also exist in asymptomatic individuals [7]. Histopathologically, MCs have been linked to endplate fissures, neovascularization, fibrovascular marrow replacement [3], pro-inflammatory mediators, and infection [5,6,8]. Neither the underlying etiopathology of MCs nor their association to LBP have been fully elucidated; however, endplate damage and disc disruption appear to be involved in a multifactorial process leading to MCs [1,8].

The endplate works as a biomechanical barrier that maintains pressure in the disc and impedes the transport of degrading molecules between the disc and the vertebrae. Damage to this barrier alters molecular transport over the endplate [9,10,11] and may increase intraosseous pressure [12] through the increased efflux of matrix molecules from the disc into the vertebral marrow [9,13]. This possible increase in pressure may in turn stimulate nociceptors in the endplates [14]. A recent study by Splendidani et al. suggested that such dynamic behavior, within a spinal segment with deteriorated endplates, can be captured with MRI. They reported an increase in MC area during spinal loading in the upright position compared to the supine position without loading, with a correlation between area increase and induced pain in the standing position [15].

Several studies have also reported that MRIs might display different tissue characteristics when conducted during spinal loading than when performed in the conventional relaxed supine position [16,17,18,19,20,21,22]. For example, MRI performed during spinal loading instantaneously display dynamic behaviors in both intervertebral discs, vertebras and endplates, indicating between-group differences in tissue characteristics in LBP patients and controls that could contribute to linking image-based features to pain [17,19]. Vertebrae with MCs have also been shown to display higher T2 times (T2-mapping) than vertebrae without [21]. However, no previous study has investigated whether the MRI signal alters in the MC itself under spinal loading. Since previous T2-mapping studies have reported instantaneous regional changes in T2 time in the disc as a response to load, a similar change could also exist in MCs. It was hypothesized that a dynamic image-based MC component exists, which was reflected as an increased T2 signal resulting from compressive spinal forces that alter the matrix architecture and/or increase the molecular flux from the disc to the vertebrae over the damaged endplate.

It is important to establish whether and how the MRI signal change in MCs during spinal loading. The aim of this study was therefore to investigate the possible effects of spinal loading on the MRI signal in MCs.

## 2. Materials and Methods

### 2.1. Study Cohort

As part of an ongoing prospective study, 100 patients were consecutively recruited to examine the general impact of spinal loading in patients with LBP. Inclusion criteria for the patients were age 20–70 years and chronic (duration ≥ 3 months) non-specific LBP. Patients were not included if they reported symptoms indicating nerve affection, previous back surgery, or claustrophobia. In the current study, all examined patients with MCs at MRI were included. A senior radiologist with >15 years’ experience in spinal MRI evaluated the presence and type of MC in each patient [3]. Background data included as part of this study were level of pain according to Visual Analog Scale (VAS) and if the patients were smokers or not. The study was approved by the regional ethics review board and conducted in accordance with the Helsinki Declaration with informed consent signed by all patients.

### 2.2. Magnetic Resonance Imaging

All patients underwent MRI examination (3T scanner/Signa, GE Healthcare) of the lumbar spine, first in a relaxed supine position (without load) and immediately thereafter during axial loading in a supine position (with load) with identical scan parameters (Table 1). The entire examination lasted approximately 40 min for each patient, including scans both with and without spinal loading. Thus, the load was applied for approximately 20 min.

MRI with load was performed using the validated Dynawell compression device [21,22]. Axial loading of about 50% of the patient’s body weight was applied using a footplate attached by side straps to a patient harness, thus simulating in the MRI scanner the loading of the lumbar spine in an upright relaxed position [21,22]. A small cushion was placed beneath the lumbar spine to prevent flexion during compression. All examinations were performed between 12:00 p.m. and 4:00 p.m.

### 2.3. Measurements

**Figure 1:**
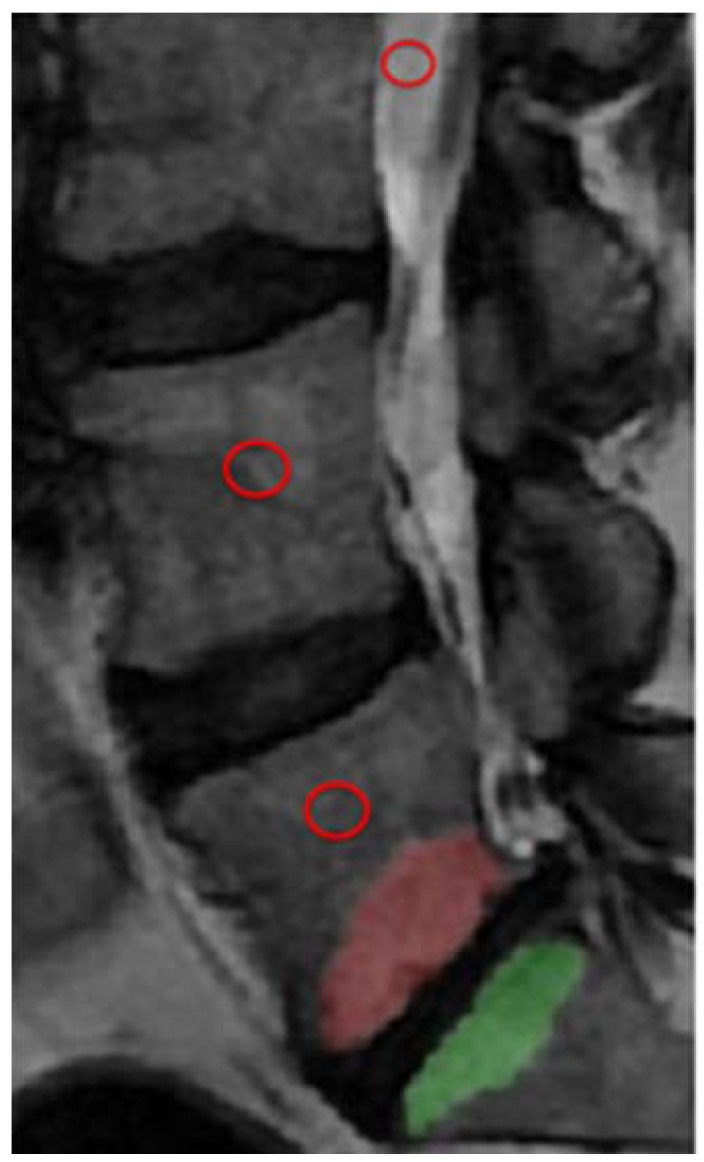
Illustration of Modic change (MC) segmentation in L5 and S1 and placement of ROIs (red circles) in cerebrospinal fluid, vertebrae without MC, and vertebra with MC.

**Figure 2:**
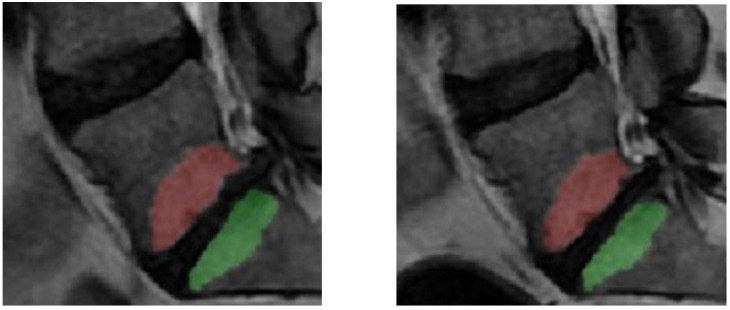
Example of segmented Modic change (MC) in L5 and in S1 at MRI without (**left**) and with (**right**) spinal loading.

A senior medical student performed the segmentation of all MCs following a supervised training phase on another dataset not included in the study. Reliability measurements, for both MC volume and MRI signal, were performed on a set of 30 MCs (the middle third of the cohort) by the medical student and by the senior radiologist, who were blinded to each other. The student then repeated the measurements after one month. Reliability measurements were performed using the intra-class correlation (ICC) coefficient. Each MC was outlined on sagittal T2-weighted (W) images using a free-hand polygonal tool (ITK-SNAP, version 3.6.0, 2017, www.itksnap.org, accessed on 7 April 2021) [23] on each of up to 11 slices where at least 25% of the vertebra was affected in either an anteroposterior or craniocaudal direction, irrespective of location within the vertebrae (anterior, posterior or lateral). From the outlined region, the volume of the entire MC was calculated, and the mean SI of the entire MC was extracted. The measures were automatically generated by the software used. When both endplates adjacent to an intervertebral disk were involved, each MC was evaluated separately (Figure 1 and Figure 2). In each vertebra affected by MC, a reference region of interest (ROI; mean size: 0.6 cm^2^) was placed as close as possible to the center of the bone tissue without MCs on the midsagittal T2W image, avoiding apparent vessels. Similar reference ROIs were also placed as close as possible to the center of vertebrae without any MCs (Figure 1). The signal intensity (SI) in the MC and in the remote ROIs was normalized to the SI in the cerebrospinal fluid (CSF). The normalized SI in the MC was then divided by the SI in the normalized remote ROIs and compared between the MRIs with and without spinal loading. MC volumes with and without loading were also compared. The CSF ROI was placed in free CSF at a level close to the vertebrae evaluated. MCs were classified by type (I–III) [3]. For mixed types, the dominant type (>50%) was registered, but the entire MC was included in the segmentation.

### 2.4. Statistics

Parameters were tested for normality. Descriptive data are presented as mean (standard deviation (SD)) for continuous variables and as absolute values and percentages for categorical variables.

Paired Student’s *t*-tests were used to compare SI and MC volumes between MRIs with and without load. ANOVA and Tukey post hoc tests was used for multiple comparisons between load-induced differences between types of MCs. Pearson’s correlation was used for MRI markers (MC volume and SI) acquired with and without spinal loading. For ICC, the absolute agreement two-way random effects model was used [24]. The level of significance was set at *p* < 0.05. Statistical analyses were performed using SPSS (IBM statistics SPSS, version 27).

## 3. Results

**Figure 3:**
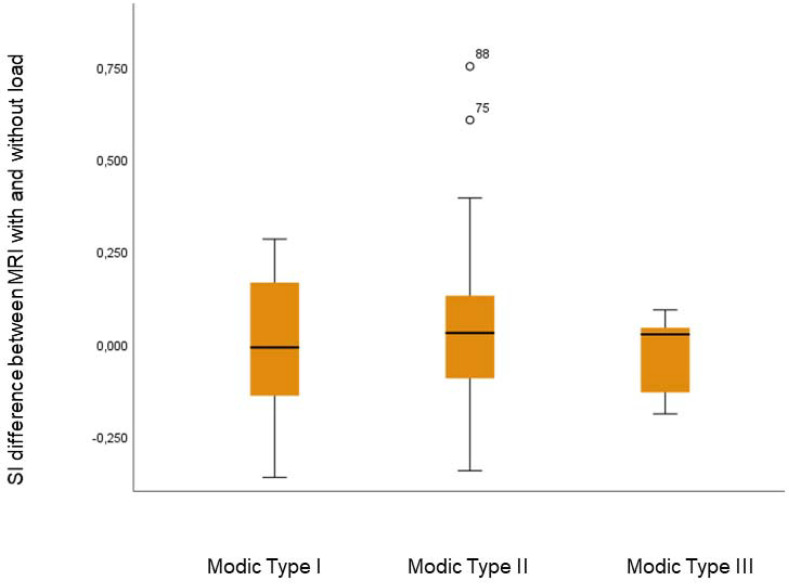
Box plot of signal intensity (SI) change between MRIs with and without load in Type I-III Modic lesions.

The presence of MCs at MRI were found in 43 individuals (23 male; mean age 45.7 years, range 29–66). The reported level of pain, in terms of VAS, in this group of LBP patients was mean 56.4 (SD 22). Only one of the individuals reported being a smoker. In these 43 individuals, a total of 94 MCs, 84% localized in L3-S1, were evaluated and distributed as follows: type I: 36.2% (n = 34), type II: 58.5% (n = 55), and type III: 5.3% (n = 5). No change in location or type was observed when load was applied. The mean (SD) SI in MCs was 1.311 (0.34) without load and 1.292 (0.30) with load. No significant SI change was detected in the MCs, either in total (mean: 0.02; 95% CI: −0.02 to 0.06; *p* = 0.308) (Table 2) or when stratified by type (0.905 > *p* < 0.151) (Table 3 and Figure 3). Spinal loading induced a higher SI in vertebral reference ROIs, both in vertebrae with MCs (mean: 0.023, *p* = 0.002) and in vertebrae without (mean 0.017, *p* = 0.02); (Figure 1). The mean (SD) SI in the reference ROIs in vertebrae with MCs was 0.445 (0.83) without load and 0.470 (0.87) with load. Corresponding figures for the reference ROIs in vertebrae not affected by MCs were 0.447 (0.1) and 0.464 (0.1), respectively.

MC volume did not change between MRIs with spinal loading (mean: 1.87cm^3^, SD: 1.5) and those without (mean: 1.92 cm^3^, SD 1.5; *p* = 0.25), even when stratifying by MC type (0.09 > *p* < 0.68); (Table 3). Correlation analyses were performed to test for any associations between induced change in MC volume and induced MC SI, but none were found (k = −0.23; *p* = 0.023), neither if correlation analysis was performed only in MCs in which spinal loading induced an increase in MC volume (k = −0.28 *p* = 0.08).

The ICC coefficient for intra-observer agreement on SI was 0.97 (95% CI: 0.90–0.99) versus 0.98 (95% CI: 0.95–0.99) for inter-observer agreement. The ICC coefficient for MC volume was 0.87 (95% CI: 0.67–0.95) for intra-observer agreement versus 0.97 (95% CI: 0.93–0.99) for inter-observer agreement.

## 4. Discussion

In this prospective MRI study investigating load-induced features in MCs, spinal loading was not found to modify either the SI or volume of MC.

It is known that the micro-architecture of vertebral bones differs between those affected by MC versus those without MC as well as between various types of MC [3,25]. Since each of the three different MC types have different tissue characteristics, it is reasonable to assume that spinal loading will impact each type differently. The bone marrow in MC type I has been shown to be replaced by vascularized granulation tissue accompanied by endplate fissuring and increased bone turnover [2,25,26]. Furthermore, in vitro studies have reported a higher ratio of erosion of the bone surface in MC type I than in type III. The precondition for a dynamic component seems therefore greater in MC type I than in, for example, MC type III, which has greater bone volume fraction and thicker trabeculae and is therefore less likely to be immediately affected by spinal dynamics [25]. However, no load-induced difference could be shown when stratifying for MC type. This study confirms the findings of Lagerstrand et al., who reported no load-induced changes in vertebrae with MCs using T2 mapping and segmentation of the entire vertebrae [20]. Our results thus contradict the findings of Splendiani et al., who reported that an increased MC area correlated with increased pain in the upright position [15], suggesting a possible diagnostic value in exploring this dynamic MC component in patients with LBP. Differences between their study and the current one could be methodological. In the current study, the volumetric segmentations included more image slices than in the Splendiani study, and the set-up was optimized to reduce potential errors due to partial volume effects and repositioning between examinations. The effect of moving from supine to standing position in the earlier study was likely larger than undergoing MRI examination both with and without load while remaining supine, as in the current study. On the other hand, spinal loading in the supine position does not reflect the true conditions of the spine in the upright position. Nevertheless, the lack of change in both MC volume and SI strengthens the results of the present study.

The biomechanical stress model describes how microtrauma of the endplate initiates a catabolic cascade of inflammatory mediators, inducing degenerative disc changes with a disturbed biochemical exchange between the disc and the vertebra [1]. This model has often been suggested as the etiopathology of MCs. Splendidani et al. explained their finding of increased MC area in the upright position through the redistribution of water from a degenerated disc to the adjacent marrow through cracks in the endplate [15]. A potential explanation of our contradictory results, which showed no image-based dynamic component in MCs, could be impaired molecular transport over the endplates due to possible endplate calcifications, sclerosis, and/or larger molecular size in solutes and restricted diffusion that could co-exist with an MC-associated inflammatory process [1,11]. Increased matrix density in a motion segment has been shown to be associated with reduced transportation of solutes [11], and it is likely that vascularized granulation tissue, inflammatory mediators, and edema, all associated with MCs, lead to a more dense matrix than in a vertebra without MC. This is supported by the finding in the recently published study by Lagerstrand et al. that investigated differences in intervertebral disc and vertebral T2-relaxation time after spinal loading [17]. Spinal loading induced less change in the motion segment with endplate changes, specifically a limited loading effect for vertebrae with MCs type I. Furthermore, they reported significantly higher T2 time values in vertebrae with MCs type I, suggesting this as a reflection of an inflammatory state, since higher T2 times represent higher content of water molecules and altered micro-architecture. Another similar study reported significant differences in SI, investigated with T2-mapping, between MRI performed with and without load in normal endplates as opposed to abnormal endplates or endplates associated with MCs [18].

The possibility of using MCs as image-based biomarkers of pain remains a matter of debate. Even if MCs, especially MC type I, are related to LBP, its use as a biomarker of individual pain is limited, since MC also exist in asymptomatic individuals [7]. A degenerative disc under biomechanical stress can produce pro-inflammatory mediators, which in turn can diffuse through the endplate and subchondral bone and generate local areas of inflammation and edema (MCs) [27]. Biomechanically induced local inflammatory changes, in general, are known to be a potential source of pain. In patients with LBP, it is common that various forms of spinal loading induce or aggravate the pain. Furthermore, concordant pain has been shown to be induced by axial loading of the spine during MRI [28]. Previous findings of instantaneous load-induced regional behavior within the disc, with differences between LBP patients and controls, strongly suggest that annular fissures are associated with pain [19,29,30]. This, combined with a lack of image-based load-induced changes in MCs, probably reflects that MCs in general are not instantaneously influenced by such biomechanical stress. Although these findings seem not to support the theory that MCs are actively involved in biomechanically induced pain, pain is a complex phenomenon. Since MCs are part of the vertebral bone, actual morphological deformation may not be evident under external stress. Radiological deformation is therefore not necessary for MCs to be pain generators; for example, the endplate itself could be affected, or nociceptors within might be stimulated by biomechanical loading. It should, however, be noted that this study examined the load-induced effect on MCs on a group, not an individual, level. Considering the relatively large range of load-induced changes (Figure 3), it cannot fully be excluded that spinal loading may modify MCs on an individual or segmental level. Theoretically, even within a certain type, MCs might represent different phenotypes; depending on the type of endplate change (sclerosis versus cracks/fissures), some may be differently affected by load. For example, it has been reported that load-induced T2-relaxation time differs between endplates with and without apparent changes [18,20]. The lack of correlation between induced change in MC volume and SI, however, makes this less likely, especially since the analyses were repeated with similar results after excluding MCs without change in volume.

**Figure 4:**
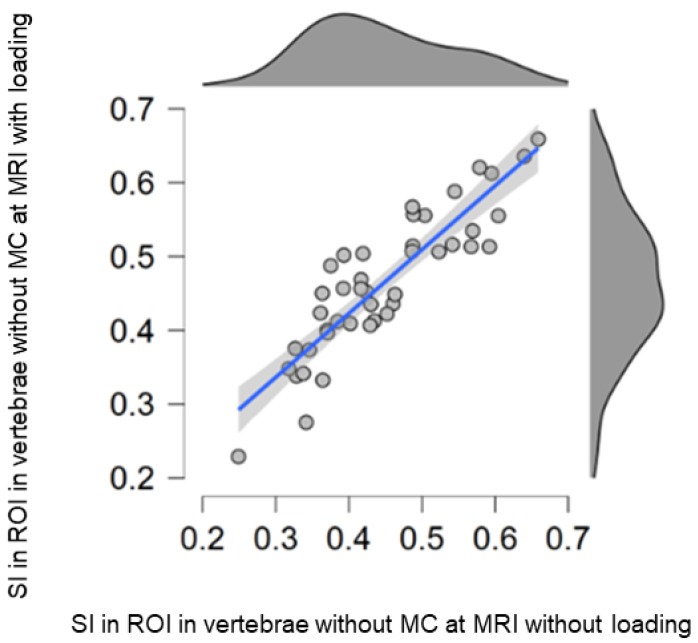
Scatterplot of the signal intensity (SI) in each reference ROI in vertebrae without Modic change (MC) at MRI with (y-axis) and without (x-axis) loading.

The current finding of minor load-induced SI increase in vertebral regions not affected by MCs could be explained by the redistribution of water molecules in the dynamic hematopoietic bone marrow [31]. Quantitative MRI techniques have long been used to phenotype bone marrow, and it is known that vertebral marrow signals depend on age, nutritional status, disease, and other factors [31,32,33]. The current study confirms a large between-individual variation in marrow signal and minor intra-individual variations. Regional signal alterations can reflect local variation in the amount of normal vertebral matrix components such as fat, bone, vessels, and hematopoietic cells [31]. Even if the induced change was significant, the effect was very small in relation to the large inter-individual variation in vertebral marrow signal (Figure 4). Different methodologies between the current study and previous studies investigating the load-induced behavior in vertebrae likely explain slightly divergent results. The few existing such studies have segmented the entire vertebrae and used T2-mapping, whereas in the current study, the entire MC was segmented. For example, Lagerstrand et al. reported a lack of significant load-induced signal change in vertebrae in both patients and controls [17], while another study reported different load-induced behavior between vertebra with and without endplate changes [20]. Present findings contribute to the understanding of MCs and demonstrate their insensitivity to different loading conditions.

### Limitations

The lack of a control group is a limitation, since significantly higher T2 values, based on T2-mapping, have been reported in the vertebrae of patients with LBP than in controls [17]. A larger cohort enabling stratification for all MC types with statistical power would be preferred. Similarly, a larger cohort would enable stratification also for MC location within the vertebrae as well as correlation between load-induced MC behavior and load-induced pain. A possible reason for the lack of significant SI change in MCs may be that the SI increase is too small relative to an already high signal at MRI without load. In addition, it cannot be excluded that prolonged load bearing would alter the MCs. As discussed above, spinal loading in a supine position does not replicate the loading conditions in an upright position; however, it has recently been shown that there is a strong correlation between spinal MRI morphology using a compression device in the supine position and spinal morphology in the standing position [22]. In addition, comparisons between this study and previous studies investigating the general load-induced effect in vertebrae might be improved if T2-mapping were used; therefore, the present results need to be confirmed in a large cohort study using quantitative sequences, such as T2-mapping, and preferably with a control group.

## 5. Conclusions

To conclude, MRIs performed with and without spinal loading showed no change in either volume or signal of MCs. The lack of radiological changes suggests that most MCs are not instantaneously influenced by biomechanical load.

## Figures and Tables

**Table 1:** MRI scan protocol.

Orientation/Sequence	TR (ms)	TE (ms)	FOV (mm)	Aqusition Matrix	Slice (mm)
sagittal T1 weighted	573	7.7	280 × 280	352 × 260	3.5
sagittal T2 weighted	3680	108	280 × 280	352 × 288	3.5

ms = milliseconds, mm = millimeters. TR = repetition time, TE = echo time, FOV = field of view.

**Table 2:** Change in Modic signal intensity and Modic volume between magnetic resonance imaging performed with and without spinal loading.

	Load-Induced Change (MRI without Load-MRI with Load)			
	MC Signal Intensity	MC Volume (cm^3^)	ROI Vertebra w MC Signal Intensity	ROI Vertebra w/o MC Signal Intensity
N	94	94	94	43
Mean	0.020	0.046	(−)0.025	(−)0.016
SD	0.187	0.387	0.07	0.09
95% CI lower	(−)0.019	(−)0.033	(−) 0.039	(−)0.064
95% CI upper	0.058	0.125	(−) 0.007	(−) 0.047

w = with; w/o = without; SD = standard deviation; CI = confidence interval, MC = Modic change, ROI = reference region of interest.

**Table 3:** Change in Modic volume and signal intensity between magnetic resonance imaging acquired with and without spinal loading for different Modic types.

Load-Induced Change (MRI without Load-MRI with Load)					
		Modic Change Signal Intensity		Modic Change Volume (cm^3^)	
Modic Type	N	Mean	SD	Mean	SD
I	34	(−)0.004	0.176	(−)0.026	0.316
II	55	0.039	0.198	0.099	0.433
III	5	(−)0.033	0.121	0.205	0.416

SD = standard deviation.

## Data Availability

The datasets used and/or analyzed during the current study are available from the corresponding author on reasonable request.

## References

[1] Dudli S., Fields A.J., Samartzis D., Karppinen J., Lotz J.C. (2016). Pathobiology of Modic changes. Eur. Spine J..

[2] Modic M.T., Masaryk T.J., Ross J., Carter J.R. (1988). Imaging of degenerative disk disease. Radiology.

[3] Modic M., Steinberg P., Ross J., Masaryk T., Carter J. (1988). Degenerative disk disease: Assessment of changes in vertebral body marrow with MR imaging. Radiology.

[4] Mera Y., Teraguchi M., Hashizume H., Oka H., Muraki S., Akune T., Kawaguchi H., Nakamura K., Tamai H., Tanaka S. (2020). Association between types of Modic changes in the lumbar region and low back pain in a large cohort: The Wakayama spine study. Eur. Spine J..

[5] Karppinen J., Koivisto K., Ketola J., Haapea M., Paananen M., Herzig K.-H., Alini M., Lotz J., Dudli S., Samartzis D. (2021). Serum biomarkers for Modic changes in patients with chronic low back pain. Eur. Spine J..

[6] Dudli S., Liebenberg E., Magnitsky S., Miller S., Demir-Deviren S., Lotz J.C. (2016). Propionibacterium acnes infected intervertebral discs cause vertebral bone marrow lesions consistent with Modic changes. J. Orthop. Res..

[7] Jensen T., Karppinen J., Sorensen J., Niinimäki J., Leboeuf-Yde C. (2008). Prevalence of vertebral endplate signal (Modic) changes and their association with non-specific low back pain—A systematic literature review. Eur. Spine J..

[8] Crockett M.T., Kelly B.S., van Baarsel S., Kavanagh E.C. (2017). Modic type 1 vertebral endplate changes: Injury, inflammation, or infection?. Am. J. Roentgenol..

[9] Rajasekaran S., Babu J.N., Arun R., Armstrong B.R.W., Shetty A.P., Murugan S. (2004). ISSLS prize winner: A study of diffusion in human lumbar discs: A serial magnetic resonance imaging study documenting the influence of the endplate on diffusion in normal and degenerate discs. Spine.

[10] Muftuler L.T., Jarman J.P., Hon J.Y., Gardner V.O., Maiman D.J., Arpinar V.E. (2015). Association between intervertebral disc degeneration and endplate perfusion studied by DCE-MRI. Eur. Spine J..

[11] Roberts S., Urban J.P., Evans H., Eisenstein S.M. (1996). Transport properties of the human cartilage endplate in relation to its composition and calcification. Spine.

[12] Yoganandan N., Larson S.J., Gallagher M., Pintar F.A., Reinartz J., Droese K. (1994). Correlation of microtrauma in the lumbar spine with intraosseous pressures. Spine.

[13] Ferguson S.J., Ito K., Nolte L.-P. (2004). Fluid flow and convective transport of solutes within the intervertebral disc. J. Biomech..

[14] Lotz J.C., Ulrich J.A. (2006). Innervation, inflammation, and hypermobility may characterize pathologic disc degeneration: Review of animal model data. JBJS.

[15] Splendiani A., Bruno F., Marsecano C., Arrigoni F., Di Cesare E., Barile A., Masciocchi C. (2019). Modic I changes size increase from supine to standing MRI correlates with increase in pain intensity in standing position: Uncovering the “biomechanical stress” and “active discopathy” theories in low back pain. Eur. Spine J..

[16] Splendiani A., Perri M., Grattacaso G., Di Tunno V., Marsecano C., Panebianco L., Gennarelli A., Felli V., Varrassi M., Barile A. (2016). Magnetic resonance imaging (MRI) of the lumbar spine with dedicated G-scan machine in the upright position: A retrospective study and our experience in 10 years with 4305 patients. Radiol. Med..

[17] Lagerstrand K., Hebelka H., Brisby H. (2019). Low back pain patients and controls display functional differences in endplates and vertebrae measured with T2-mapping. Eur. Spine J..

[18] Hebelka H., Miron A., Kasperska I., Brisby H., Lagerstrand K. (2018). Axial loading during MRI induces significant T2 value changes in vertebral endplates—A feasibility study on patients with low back pain. J. Orthop. Surg. Res..

[19] Hebelka H., Torén L., Lagerstrand K., Brisby H. (2018). Axial loading during MRI reveals deviant characteristics within posterior IVD regions between low back pain patients and controls. Eur. Spine J..

[20] Lagerstrand K., Brisby H., Hebelka H. (2021). Associations between high-intensity zones, endplate, and Modic changes and their effect on T2-mapping with and without spinal load. J. Orthop. Res.^®^.

[21] Willen J., Danielson B. (2001). The diagnostic effect from axial loading of the lumbar spine during computed tomography and magnetic resonance imaging in patients with degenerative disorders. Spine.

[22] Charoensuk J., Laothamatas J., Sungkarat W., Worapruekjaru L., Hooncharoen B., Chousangsuntorn K. (2021). Axial loading during supine MRI for improved assessment of lumbar spine: Comparison with standing MRI. Acta Radiol..

[23] Yushkevich P.A., Piven J., Hazlett H.C., Smith R.G., Ho S., Gee J.C., Gerig G. (2006). User-guided 3D active contour segmentation of anatomical structures: Significantly improved efficiency and reliability. Neuroimage.

[24] Shrout P.E., Fleiss J.L. (1979). Intraclass correlations: Uses in assessing rater reliability. Psychol. Bull..

[25] Perilli E., Parkinson I.H., Truong L.-H., Chong K.C., Fazzalari N.L., Osti O.L. (2015). Modic (endplate) changes in the lumbar spine: Bone micro-architecture and remodelling. Eur. Spine J..

[26] Järvinen J., Niinimäki J., Karppinen J., Takalo R., Haapea M., Tervonen O. (2020). Does bone scintigraphy show Modic changes associated with increased bone turnover?. Eur. J. Radiol. Open.

[27] Nguyen C., Jousse M., Poiraudeau S., Feydy A., Rannou F. (2017). Intervertebral disc and vertebral endplate subchondral changes associated with Modic 1 changes of the lumbar spine: A cross-sectional study. BMC Musculoskelet. Disord..

[28] Hebelka H., Brisby H., Hansson T. (2014). Comparison between pain at discography and morphological disc changes at axial loaded MRI in patients with low back pain. Eur. Spine J..

[29] Torén L., Lagerstrand K., Waldenberg C., Brisby H., Hebelka H. (2020). MRI during Spinal Loading Reveals Intervertebral Disc Behavior Corresponding to Discogram Findings of Annular Fissures and Pain Provocation. Spine.

[30] Waldenberg C., Hebelka H., Brisby H., Lagerstrand K.M. (2019). Differences in IVD characteristics between low back pain patients and controls associated with HIZ as revealed with quantitative MRI. PLoS ONE.

[31] Berg B.C.V., Lecouvet F.E., Galant C., Simoni P., Malghem J. (2009). Normal Variants of the Bone Marrow at MR Imaging of the Spine. Seminars in Musculoskeletal Radiology.

[32] Karampinos D.C., Ruschke S., Dieckmeyer M., Diefenbach M., Franz D., Gersing A.S., Krug R., Baum T. (2018). Quantitative MRI and spectroscopy of bone marrow. J. Magn. Reson. Imaging.

[33] Krug R., Joseph G.B., Han M., Fields A., Cheung J., Mundada M., Bailey J., Rochette A., Ballatori A., McCulloch C.E. (2019). Associations between vertebral body fat fraction and intervertebral disc biochemical composition as assessed by quantitative MRI. J. Magn. Reson. Imaging.

